# Pterostilbene Changes Epigenetic Marks at Enhancer Regions of Oncogenes in Breast Cancer Cells

**DOI:** 10.3390/antiox10081232

**Published:** 2021-07-30

**Authors:** Sadaf Harandi-Zadeh, Cayla Boycott, Megan Beetch, Tony Yang, Benjamin J. E. Martin, Kevin Ren, Anna Kwasniak, John H. Dupuis, Katarzyna Lubecka, Rickey Y. Yada, LeAnn J. Howe, Barbara Stefanska

**Affiliations:** 1 Food, Nutrition and Health Program, Faculty of Land and Food Systems, University of British Columbia, Vancouver, BC V6T 1Z4, Canada; sadafh2005@yahoo.com (S.H.-Z.); cayla.boycott@ubc.ca (C.B.); beet0013@umn.edu (M.B.); tyang85@mail.ubc.ca (T.Y.); renkevin@student.ubc.ca (K.R.); anna.kwasniak@ubc.ca (A.K.); john.dupuis@ubc.ca (J.H.D.); r.yada@ubc.ca (R.Y.Y.); 2 Department of Biochemistry and Molecular Biology, University of British Columbia, Vancouver, BC V6T 1Z3, Canada; benjamin_martin@hms.harvard.edu (B.J.E.M.); ljhowe@mail.ubc.ca (L.J.H.); 3 Department of Biological Chemistry and Molecular Pharmacology, Harvard Medical School, Boston, MA 02115, USA; 4 Department of Biomedical Chemistry, Medical University of Lodz, 92-215 Lodz, Poland; katarzyna.lubecka@umed.lodz.pl

**Keywords:** pterostilbene, enhancer, DNA methylation, DNMT3B, H3K36 trimethylation, oncogenes, chromatin, cancer

## Abstract

Epigenetic aberrations are linked to sporadic breast cancer. Interestingly, certain dietary polyphenols with anti-cancer effects, such as pterostilbene (PTS), have been shown to regulate gene expression by altering epigenetic patterns. Our group has proposed the involvement of DNA methylation and DNA methyltransferase 3B (DNMT3B) as vital players in PTS-mediated suppression of candidate oncogenes and suggested a role of enhancers as target regions. In the present study, we assess a genome-wide impact of PTS on epigenetic marks at enhancers in highly invasive MCF10CA1a breast cancer cells. Following chromatin immunoprecipitation (ChIP)-sequencing in MCF10CA1a cells treated with 7 μM PTS for 9 days, we discovered that PTS leads to increased binding of DNMT3B at enhancers of 77 genes, and 17 of those genes display an overlapping decrease in the occupancy of trimethylation at lysine 36 of histone 3 (H3K36me3), a mark of active enhancers. We selected two genes, *PITPNC1* and *LINC00910*, and found that their enhancers are hypermethylated in response to PTS. These changes coincided with the downregulation of gene expression. Of importance, we showed that 6 out of 17 target enhancers, including *PITPNC1* and *LINC00910*, are bound by an oncogenic transcription factor OCT1 in MCF10CA1a cells. Indeed, the six enhancers corresponded to genes with established or putative cancer-driving functions. PTS led to a decrease in OCT1 binding at those enhancers, and OCT1 depletion resulted in *PITPNC1* and *LINC00910* downregulation, further demonstrating a role for OCT1 in transcriptional regulation. Our findings provide novel evidence for the epigenetic regulation of enhancer regions by dietary polyphenols in breast cancer cells.

## 1. Introduction

Breast cancer is the most commonly diagnosed malignancy and the second leading cause of cancer-related deaths among women [1]. Epigenetic alterations are found frequently in breast cancer and are suggested to drive a number of sporadic cases [2]. Epigenetics refers to the control of gene expression without changes to the DNA sequence. The epigenetic code consists of DNA methylation, covalent histone modifications, noncoding RNA mechanisms, and chromatin remodeling complexes [3,4]. All components of the epigenetic machinery are important to influence gene expression; however, DNA methylation mainly occurs on the cytosine of CpG dinucleotides and is catalyzed by specific enzymes, namely, DNA methyltransferases (DNMTs), and they are particularly considered to stabilize gene transcription [3,4,5]. DNA methylation reactions are thought to provide stable, long-term regulation of gene expression over time [6], while at the same time are considered to be dynamic and responsive to external exposures [7]. During carcinogenesis, perturbations in the DNA methylation machinery occur, leading to aberrant DNA methylation patterns in sequences that are involved in the regulation of gene transcription, which subsequently affects the expression of genes related to cell differentiation, proliferation, metastasis, and other biological processes [8]. For decades, researchers were reporting on aberrant DNA methylation levels within promoters of genes while the role and regulation of other regulatory regions, e.g., enhancers, silencers, or insulators, were largely disregarded [9]. Studies over the last 20 years clearly demonstrate that the DNA methylation status of enhancer regions may impact their regulatory role in addition to histone marks [10].

Enhancers are usually 30–200 base pairs long and may be located upstream or downstream of their target genes [9,11]. The activity of enhancers is highly dependent on a cell type, physiological stimuli, and a specific developmental time point. These distant regulatory elements increase the likelihood of gene transcription by interacting with their target gene promoters via looping of DNA in association with transcription factors, RNA polymerase, and other cofactors [9,11]. Another way through which enhancers can impact the expression of target genes is through their own transcription and generating enhancer RNA (eRNA), which positively correlates with mRNA synthesis of the target genes [12]. Several classes of enhancers have been described, including active, intermediate, and poised, that are characterized by distinct histone marks [13]. For example, active enhancers are rich in acetylation of lysine 27 of histone H3 (H3K27ac), whereas poised enhancers are marked by trimethylation of H3K27 (H3K27me3) [9,13]. Of importance, H3K36me3 has been shown as a unique mark of active enhancers, distinguishing active from intermediate or poised enhancers [13]. It is crucial to emphasize that H3K36me3 is a mark generally associated with actively transcribed genes and transcriptional elongation; thereby, it is also present at other regions relevant to transcription [13]. Apart from distinct patterns of histone covalent modifications, active enhancers display less DNA methylation [10,14]. DNA hypomethylation was also proposed as a signature of eRNA-generating enhancers, thus active enhancers [15]. While our knowledge about the regulation of enhancers is constantly increasing, approaches to reverse aberrant enhancer activity in disease remain undiscovered.

Remarkably, the last decade of research has shown epigenetic activities exerted by compounds with polyphenolic structure, including stilbenoid polyphenols [16,17,18,19,20,21,22], accompanied by their potent anti-cancer effects [23,24,25]. Studies have shown loci-specific changes in DNA methylation and reversal of cancer-like DNA methylation patterns [16,17,18,19,20,21]. Among these compounds, Pterostilbene (PTS), a naturally present stilbenoid in the diet (e.g., blueberries, grapes, cranberries, peanuts, pistachios, cocoa), stands out due to its high bioavailability [26,27]. We and others previously observed changes in DNA methylation at candidate gene loci, including *PTEN*, *APC*, and *BRCA1* in human cancer cells exposed to PTS or its analog resveratrol (RSV) [16,17,18]. These reports were strengthened by our genome-wide study to demonstrate remodeling of the DNA methylation patterns in breast cancer cells exposed to stilbenoids [20]. We observed increases and decreases in DNA methylation levels at thousands of CpG loci. These findings laid the framework for subsequent work to identify mechanisms underlying stilbenoid-mediated changes in loci-specific DNA methylation [20,28]. For the first time, we demonstrated that stilbenoid-mediated downregulation of an oncogene, *MAML2*, which is an activator of the NOTCH signaling pathway, is accompanied by increased DNA methylation at the *MAML2* enhancer region [20]. DNMT3B was found to bind the *MAML2* enhancer upon treatment with stilbenoids, suggesting a role for this DNA methyltransferase in methylating the enhancer [20]. We further showed that one-third of hypermethylation events in response to stilbenoids occur in gene bodies, and the majority of those loci within gene bodies are located in gene enhancers [20]. In the current study, we have therefore investigated how stilbenoids, specifically PTS, impact epigenetic marks at enhancer regions in MCF10CA1a breast cancer cells. To better understand the role of DNMT3B binding, DNA methylation, and histone modifications at enhancer regions in stilbenoids-mediated downregulation of gene transcription, we have performed chromatin immunoprecipitation (ChIP) followed by next-generation sequencing to analyze binding events of DNMT3B and occupancy of H3K36me3 at a genome-wide scale. We then elucidated whether altered DNMT3B and H3K36me3 enrichment are linked to changes in transcription factor binding and gene transcriptional activity.

## 2. Materials and Methods

### 2.1. Cell Culture and Treatment with Pterostilbene (PTS)

Human breast cancer cell line, MCF10CA1a, used in this study, was cultured in DMEM/F12 (1:1) medium (Gibco, Waltham, MA, USA) supplemented with 5% horse serum (Gibco, Waltham, MA, USA), 1 U/mL penicillin, and 1 μg/mL streptomycin (Gibco, Waltham, MA, USA), and grown in a humidified atmosphere of 5% carbon dioxide at 37 °C. The cell line was derived from tumor xenografts of MCF10A cells transformed with constitutively active Harvey-ras oncogene and represented lowly differentiated malignant tumor with high invasive potential. The cell line was obtained from Dr. Dorothy Teegarden, Purdue University, IN, USA. Pterostilbene (PTS, Cayman Chem., Ann Arbor, MI, USA) was resuspended in ethanol, and 10 mM stock solution was stored at −20 °C. Next, cells were plated at a density of 2–3 × 10^5^ per 10 cm tissue culture dish 24 h prior to treatment with PTS. Freshly diluted PTS was prepared prior to adding to the culture medium. Cells were treated with 7 μM PTS for 4 days. Cells were then split at 1:50 ratio and exposed to the compound for additional 4 days (9-day exposure). The concentration of 7 μM was determined in our previous studies as the IC50 concentration for 9-day treatment [20,29]. Please note that the IC50 dose reduces cell growth by 50% and leads to not more than 10% of dead cells compared to vehicle-treated cells (ethanol used as a solvent for PTS).

### 2.2. Chromatin Immunoprecipitation (ChIP) Sequencing (ChIP-Seq)

Chromatin immunoprecipitation was performed as previously described [20,30]. In brief, cells representing three biological replicates were fixed with 1% formaldehyde and incubated at 37 °C for 15 min. Fixed cells were lysed, followed by 15 s intervals of sonication (amplitude 40; Qsonica Q55), interspaced with 45 s rest, for 20 cycles on ice. Lysates were pre-cleared with protein G agarose and centrifuged to collect supernatants, which were divided into three fractions. One fraction was maintained as input. The second fraction served as negative control and was incubated overnight at 4 °C with IgG non-specific antibody (negative control, Santa-Cruz Biotechnology, Dallas, TX, USA). The third fraction was incubated overnight at 4 °C with the following antibodies: anti-DNMT3B rat antibody (Millipore, Burlington, VT, USA, MABE305), anti-trimethyl-Histone H3 Lys36 rabbit antibody (H3K36me3, Millipore, Burlington, VT, USA, ABE305), or anti-OCT1 mouse antibody (Millipore, Burlington, VT, USA, MAB5434). The following day, the DNA bound to antibodies was washed, eluted, and the antibodies were degraded using proteinase K treatment. Input and purified ChIP DNA were used to generate sequencing libraries using NEBNext Ultra DNA Library Prep Kit for Illumina reagents according to the manufacturer’s protocol. ChIP libraries were sequenced using PE150bp reads in HiSeq2500, as described previously [31,32].

### 2.3. Analysis of ChIP-Seq Data

As described previously, ChIP-seq data were analyzed using Bioconductor tool in R [31,32]. Adapter sequences were trimmed from sequencing reads using cutadapt [33], and reads were aligned to the GRCh37/hg19 human reference genome using the Burrows–Wheeler Aligner [34]. Duplicate and low-quality reads were filtered out. MACS2 peak calling software was used to identify distinct patterns of enrichment in control-treated versus PTS-treated samples and to generate fold enrichment tracks [35,36]. Briefly, the callpeak function was used to identify peaks in the control or treated samples, using the pooled ChIP-seq inputs as the background control and using 200 bp as the estimated fragment size and an effective genome size of 2,700,000,000. The broad option was used for calling H3K36me3 peaks. Differential peaks were called using the bdgdiff function. Fold enrichment over input tracks was generated from pileup tracks using the bdgcmp function and the -m FE option. To visualize accurate representation of our results, read-extended bigwig files of our obtained genomic data were implemented in the genome browser. Lastly, ChIPSeeker Bioconductor package was used to associate the identified peaks to their target genes. The ChIP quality control (CHIPQC) Bioconductor package calculated ChIP-seq specific quality metric for each sample and input in our experiment. CHIPQC further identifies both fragment-length peak and a read-length peak based on cross-coverage around the centers of binding sites. CHIPQC was used to both measure inequality of coverage across the genome via standardized Standard Deviation (SSD) and assess distribution of ChIP-seq signal over genomic regions.

To assign peaks to chromatin states, broad ChromHMM data from human mammary epithelial cells (HMEC) were used [37]. Peaks could correspond to the following chromatin states: active, weak, or poised promoters, strong or weak enhancers, putative insulators, active or weak transcription, Polycomb-repressed regions or heterochromatin. Active promoters, strong enhancers, and active transcription regions are linked to high expression levels, with the latter state determined based on the enrichment of histone marks along transcripts. The ChIP-seq data are available from Gene Expression Omnibus (accession number: GSE175639).

### 2.4. DNA Isolation and Pyrosequencing

DNA was isolated using standard phenol:chloroform extraction protocol. DNA bisulfite conversion was performed as previously described [20]. HotStar Taq DNA polymerase (Qiagen, Hilden, Germany) and biotinylated primers were used to amplify bisulfite converted DNA with primers specific for studied gene regulatory regions (please see Supplementary Table S1 for primer sequences). Pyrosequencing of the biotinylated DNA strands was performed in the PyroMark Q48 Autoprep instrument (Qiagen, Hilden, Germenay), as previously described [38]. Percentage of methylation at a single CpG site resolution was calculated using PyroMark Q48 software.

### 2.5. RNA Isolation, cDNA Synthesis and QPCR

TRIzol reagent (Invitrogen, Waltham, MA, USA) was used to isolate total RNA from MCF10CA1a cells treated with 7 μM PTS for 9 days. cDNA was synthesized using 1 μg of total RNA as a template and 20 U of AMV reverse transcriptase (Roche Diagnostics, Basel, Switzerland). The QPCR reaction was carried out in CFX96 Touch Real-Time PCR Detection System (Bio-Rad, Hercules, CA, USA) using 2 μL of cDNA, 10 μL of SsoFast EvaGreen Supermix (Bio-Rad), and 400 nM forward and reverse primers (please see Supplementary Table S1 for primer sequences), in a final volume of 20 μL. The amplification reactions were performed in biological triplicate under the following conditions: denaturation at 95 °C for 10 min, amplification for 40 cycles at: 95 °C for 10 s, annealing temperature for 10 s, 72 °C for 10 s; and final extension at 72 °C for 10 min. Genes transcript levels (quantification of the gene expression level) were quantified using the CFX Maestro Software (Bio-Rad, Hercules, CA, USA) with standard curve-based analysis. Relative gene expression levels of target genes are presented as gene of interest/GAPDH (reference gene).

### 2.6. Statistical Analysis

Unpaired *t*-test with two-tailed distribution was used for statistical analysis of QPCR and pyrosequencing data. Each value represents the mean ± S.D. of three independent experiments (biological replicates). The results were considered statistically significant when *p* < 0.05.

## 3. Results

### 3.1. Overview of DNMT3B Binding in Highly Invasive MCF10CA1a Breast Cancer Cells in Response to Pterostilbene (PTS)

**Figure 1:**
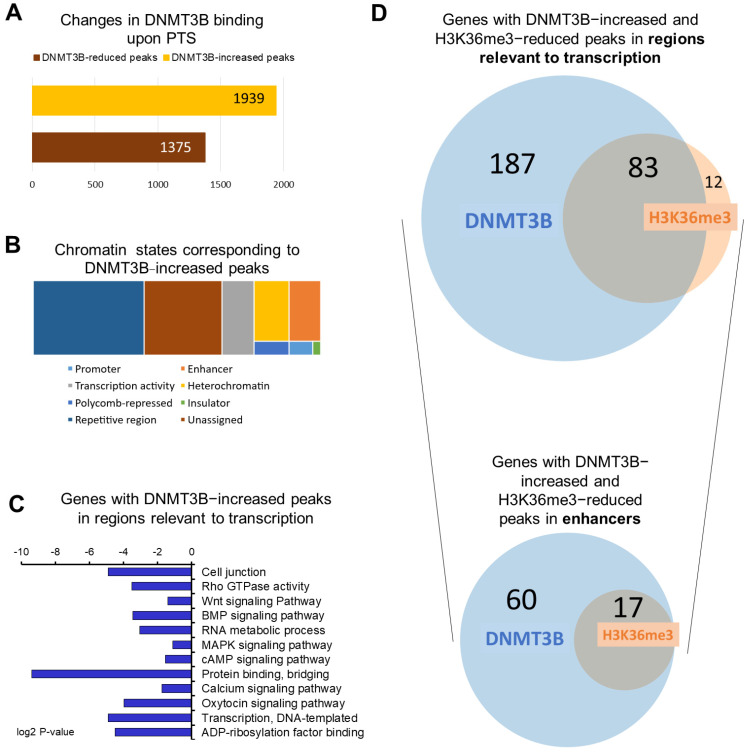
Overview of genome−wide changes of DNMT3B binding in response to PTS. (**A**) A bar chart depicting the number of changes in DNMT3B binding in MCF10CA1a breast cancer cells treated with 7 μM PTS for 9 days. DNMT3B binding was assessed by ChIP sequencing. (**B**) Chromatin states associated with DNMT3B-increased peaks were determined using Broad ChromHMM data from human mammary epithelial cells (HMEC) available on USCS Genome Browser (hg19). Peaks could correspond to active, weak, or poised promoters, strong or weak enhancers, putative insulators, active or weak transcription, Polycomb−repressed regions, heterochromatin, or repetitive regions. (**C**) The 270 genes with DNMT3B-increased peaks in regions relevant to transcription upon PTS (i.e., promoters, enhancers, insulators, Polycomb-repressed regions, or heterochromatin) were subjected to Gene Ontology (GO) function and Kyoto Encyclopedia of Genes and Genomes (KEGG) pathway analysis using DAVID Knowledgebase. (**D**) Venn diagrams present an overlap between genes with DNMT3B−increased and H3K36me3-reduced peaks that were located in regions relevant to transcription and specifically in enhancer regions. The proportionally scaled Venn diagrams were generated using tools on www.stefanjol.nl/venny (accessed on 28 May 2021).

To understand a possible functional role of DNMT3B in mediating epigenetic effects of stilbenoid polyphenols, we performed ChIP for DNMT3B in invasive MCF10CA1a breast cancer cells upon 9-day treatment with PTS at 7 μM concentration, followed by next-generation sequencing. Upon analysis of DNMT3B ChIP-seq data, we identified statistically significant changes in DNMT3B binding in 3314 peaks throughout the genome. Of those peaks, 1939 peaks were enriched with DNMT3B upon PTS (Figure 1A).

Using the Broad ChromHMM track associated with human mammary epithelial cells (HMEC) available on the USCS Genome Browser, DNMT3B-increased peaks were annotated to corresponding chromatin states. The peaks were mostly located in repetitive elements (38.5% of peaks), which is in accordance with the well-established DNMT3B function in transcriptional repression of these elements, crucial for genomic stability [39]. While 27% of peaks were located in regions with unspecified chromatin state, the remaining 665 peaks were found in regions important for regulation of gene transcription, including promoters, enhancers, insulators, Polycomb-repressed regions, or heterochromatin. (Figure 1B).

Using gene ontology (GO) and KEGG tools in the DAVID knowledgebase database, we performed functional and signaling pathway analyses of genes that contained at least one of the 665 DNMT3B-enriched peaks within regions relevant to transcription (Figure 1C). We identified 270 unique genes in this group. We found that these genes are implicated in signaling pathways commonly upregulated in cancer (Wnt, MAPK, BMP, mTOR, NOTCH, PI3K/Akt), in DNA replication, recombination and repair, cell junction, actin cytoskeleton, regulation of transcription, and calcium ion transmembrane transport (Figure 1C). Thereafter we refer to the 270 genes as DNMT3B-enriched target genes.

A thorough analysis of the DNMT3B-enriched target genes revealed candidates with oncogenic functions, including *NOTCH2NL*, *PANX1*, *PVT1*, and *JAK2*. Of note, *NOTCH2NL* is an oncogene activating Notch signaling by direct interaction with NOTCH2, thereby promoting proliferation and self-renewal [40]. *PANX1* overexpression has been associated with a worse prognosis in breast and liver cancer patients, which mechanistically is linked to PANX1-dependent enhancement of epithelial–mesenchymal transition and thus cell invasion [41,42]. *PVT1* is a long noncoding RNA that is commonly overexpressed in breast cancer and has been implicated in the regulation of MYC oncogene [43,44], while *JAK2* is a tyrosine kinase activating cancer-driving JAK/STAT signaling pathway [45].

Among 665 peaks assigned to chromatin states that are relevant to gene transcription, 201 peaks were located within promoters or enhancers, both of which are the core cis-regulatory elements. Interestingly, the majority of those 201 peaks are in enhancer regions (i.e., 173 peaks corresponding to 77 unique genes). The 77 genes with enhancers that gained DNMT3B binding upon PTS fell into several functional categories: (1) oncogenes such as *PITPNC1* [46], *NOTCH2NL* [40], *TNNT2* [47], and *ZP4* [48], (2) long noncoding RNAs (lncRNAs) such as *DANT2*, *LINC00910*, and *LOC102724511*, (3) microRNAs *miR4477A* and *miR4477B*, (4) small noncoding RNAs *RNVU1-18* and *SNAR-A14*), (5) pseudogenes *LOC100130331*, *LOC102724580*, and (6) epigenetic regulator *SMARCA4*, which is the ATPase of the chromatin-remodeling SWI/SNF complexes [49]. Many of those genes are linked to functions and processes that drive cancer. For instance, serine/threonine protein kinase *NLK* was demonstrated to localize in the nucleus of breast cancer cells and protect the cells from apoptosis [50]. The tumorigenic function of the *SREBF1* transcription factor, which was found to be overexpressed and positively associated with metastasis and poor prognosis in breast cancer patients, has been linked to oncogenic activation of the PI3K/AKT/mTOR signaling pathway [51]. *PITPNC1* has been shown to be overexpressed in metastatic breast, colon, and melanoma cancers [46], and *SMARCA4* is a marker of poor prognosis in many types of cancer [49]. We also identified a group of noncoding RNAs with DNMT3B-enriched enhancers (e.g., *LINC00910*). Although understanding of functions of noncoding RNAs in carcinogenesis is limited, there is evidence indicating that noncoding RNAs impact a wide range of biological processes such as metabolism [52], immune response [53], and development [54], all of which have been found to be dysregulated during carcinogenesis.

### 3.2. H3K36me3 Occupancy Is Reduced at Regions Enriched with DNMT3B in Response to PTS

**Figure 2:**
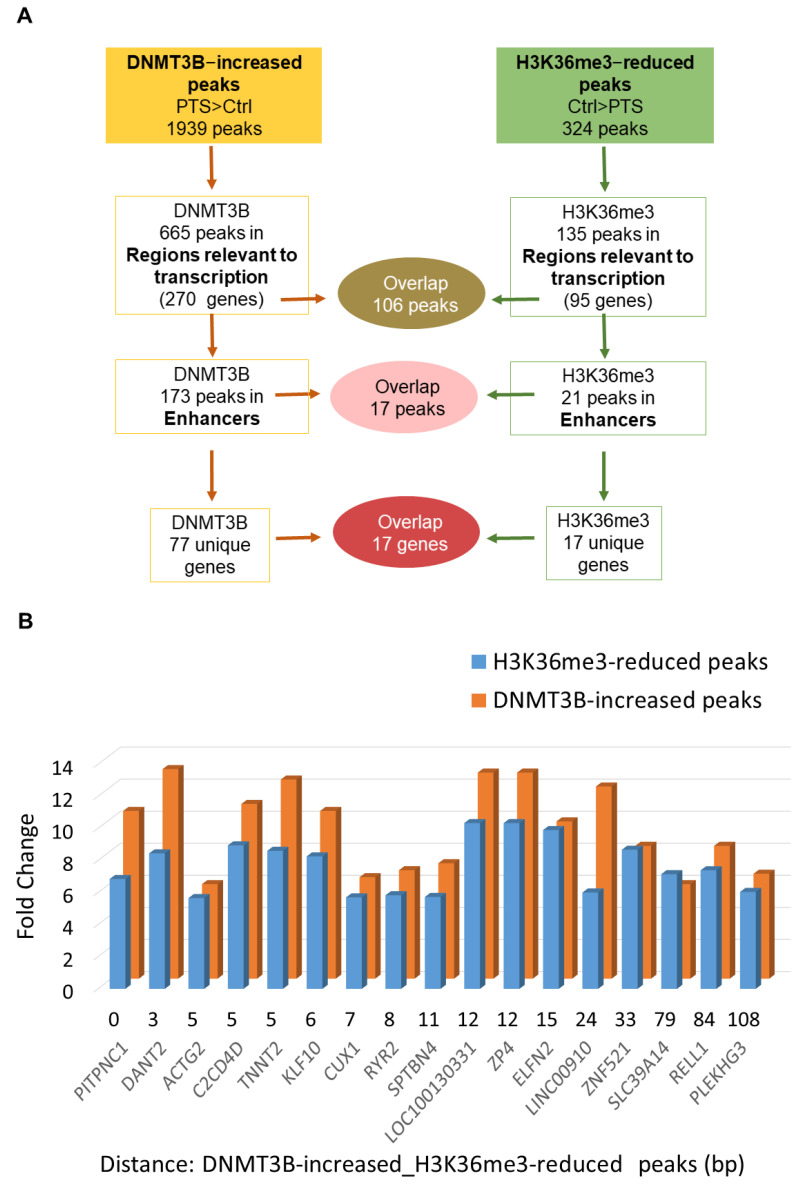
Characterization of regions and genes that contain overlapping DNMT3B-increased and H3K36me3-reduced peaks in response to PTS. (**A**) Schematic of the analysis of peaks and genes associated with DNMT3B-increased and H3K36me3-reduced peaks in MCF10CA1a breast cancer cells treated with 7 μM PTS for 9 days. The overlap between peaks located within regions relevant to transcription (i.e., promoters, enhancers, insulators, Polycomb-repressed regions, or heterochromatin) and specifically enhancers is depicted along with the number of corresponding genes. (**B**) The 17 overlapping peaks located in enhancer regions and the corresponding genes are shown in the bar chart that computes a difference (fold change) in DNMT3B binding and H3K36me3 enrichment between PTS-treated and control cells (vehicle-treated, ethanol), along with the distance between DNMT3B-increased and H3K36me3-reduced peaks in a given gene. The presented results are based on ChIP-seq analyses.

In order to test whether DNMT3B binding is linked to changes in the activity of enhancers, we performed ChIP-seq with antibodies recognizing H3K27ac, a histone mark at active enhancers, in our preliminary experiment. However, we did not detect any apparent changes in H3K27ac upon PTS. Therefore, we proceeded with assessing PTS impact on the distribution of H3K36me3, which is a unique mark that can distinguish active enhancers from intermediate and poised enhancers and is associated with transcriptional activity [13]. Using ChIP-seq, we found 673 peaks, which showed statistically significant differential enrichment with H3K36me3 in response to PTS. Among those peaks, there were 324 peaks with a reduced enrichment in cells treated with PTS, as compared with vehicle-treated cells. The analysis of the chromatin states showed that 135/324 peaks are located in regions relevant to transcription and correspond to 95 unique genes. Our hypothesis, formulated based on our previous study [20], was that DNMT3B binds enhancers of oncogenes, catalyzes DNA methylation, and reduces the activity of enhancers upon PTS, which consequently downregulates gene expression. Thus, we focused on finding an overlap between genes with DNMT3B-increased peaks and genes with H3K36me3-reduced peaks, with the latter mark indicating reduced activity of an enhancer in a given gene. As shown in Figure 1D, we identified 83 genes that had reduced H3K36me3 mark and increased DNMT3B binding within regions relevant to transcription. Importantly, among genes with H3K36me3-reduced peaks, 17 genes were associated with changes in enhancers, and all 17 genes were also found within genes containing DNMT3B-increased peaks (Figure 1D and Figure 2A). For all of the 17 genes, reduced H3K36me3 occupancy and increased DNMT3B binding were detected within 200 bp in the same enhancer region (Figure 2B). We next focused on the 17 genes for further analysis and refer to these genes as ‘candidate genes with epigenetically targeted enhancers in response to PTS’ (Figure 2B). Among them, there were known oncogenes, *PITPNC1* [46], *TNNT2* [47], and *ZP4* [48], and lncRNAs with a putative oncogenic role, such as *DANT2* and *LINC00910*, that we mentioned in the previous paragraph. We also found several genes that may function as oncogenes within the context of breast cancer. For example, *CUX1* encodes for a transcription factor that has been demonstrated to bind to the Snail promoter, which ultimately leads to increased epithelial-to-mesenchymal transition (EMT), tumor migration, and invasion [55]. Recently, active *CUX1* has been shown to be upregulated in triple-negative breast cancer, and upon knockdown of *CUX1*, there was increased estrogen receptor ERα and drug sensitivity [56]. Another candidate, *RYR2,* encodes for a ryanodine receptor, a component of a calcium channel, and it has been previously demonstrated that altered cellular calcium homeostasis contributes to the EMT in breast cancer [57,58]. Indeed, it was described that upon the EMT of MDA-MB-468 breast cancer cells, there was a drastic increase in *RYR2*, indicating that this receptor may play an important role in the process.

### 3.3. Candidate Genes with Enhancers That Are Characterized by Increased DNMT3B and Reduced H3K36me3 Occupancy Are Hypermethylated and Downregulated upon Exposure to PTS

**Figure 3:**
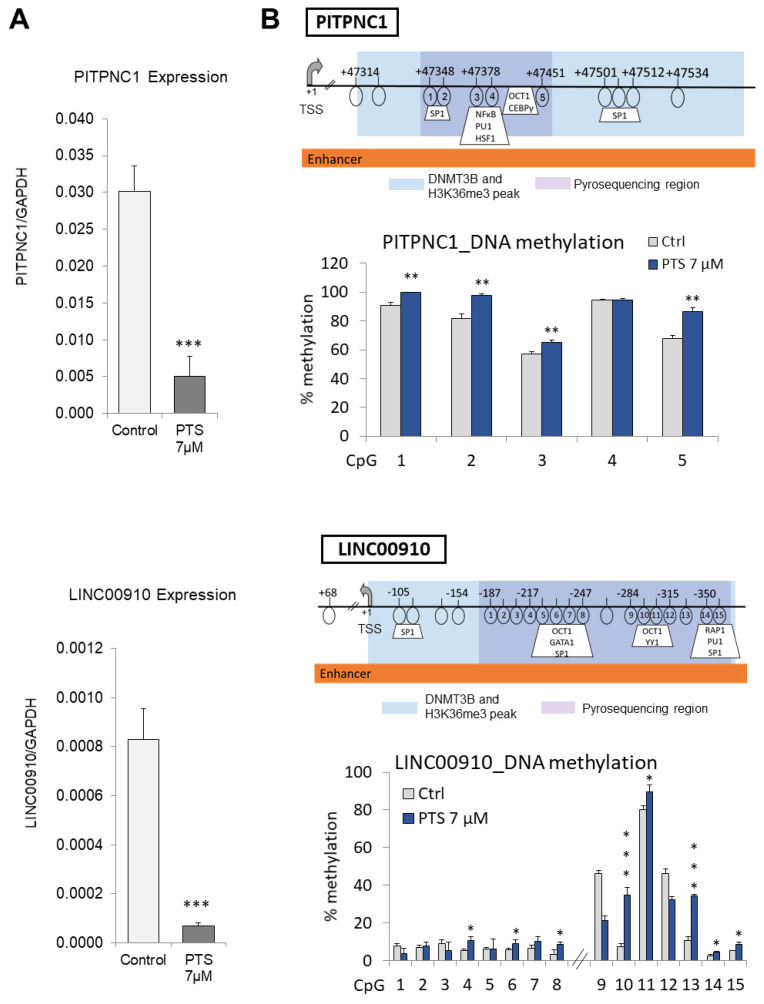
Increased DNMT3B binding and reduced H3K36me3 occupancy is associated with *PITPNC1* and *LINC00910* downregulation and hypermethylation of the enhancer regions in response to PTS in breast cancer cells. (**A**) *PITPNC1* and *LINC00910* expression upon 9-day treatment of MCF10CA1a breast cancer cells with 7 μM PTS as determined by QPCR. (**B**) Average methylation status of CpG sites within *PITPNC1* and *LINC00910* enhancer regions as determined by pyrosequencing in MCF10CA1a breast cancer cells treated for 9 days with 7 μM PTS. Gene maps show transcription start site (TSS) indicated by +1, transcription factors predicted using Transfac, and pyrosequenced CpG sites circled and numbered. The blue-shaded fragment in the gene map represents the DNMT3B-increased and H3K36me3-reduced overlapping peaks. The purple-shaded regions refer to a region tested in pyrosequencing. All results represent mean ± SD of three independent experiments; *** *p* < 0.001, ** *p* < 0.01, * *p* < 0.05.

Of the 17 ‘candidate genes with epigenetically targeted enhancers in response to PTS’, we selected two genes, *PITPNC1* and *LINC00910,* for further analyses. In our selection, we took into account the magnitude of differential binding (fold change) and the highest proximity, within less than 30 base pairs, between peaks with increased binding of DNMT3B and decreased enrichment of H3K36me3 (Figure 2B). Another important criterion was the biological functions of the genes. As mentioned before, *PITPNC1* is a reported oncogene in breast cancer [46], and *LINC00910* is a putative oncogenic noncoding RNA. To test whether a decrease in enhancer activating H3K36me3 mark impacts gene transcription, we first assessed gene expression of *PITPNC1* and *LINC00910* following 9-day treatment of MCF10CA1a breast cancer cells with 7 μM PTS. Using QPCR, we detected that PTS treatment led to a robust decrease in expression of *PITPNC* by 83% and *LINC00910* by 92%, compared to control cells (Figure 3A).

Gene downregulation and DNMT3B binding could suggest that DNA methylation within the enhancers of the tested genes underlies gene silencing. We next measured the DNA methylation status of *PITPNC1* and *LINC00910* by pyrosequencing. Gene maps show the exact positions of CpG sites in regions of differential occupancy of DNMT3B and H3K36me3 relative to the transcription start site (+1, TSS) (Figure 3B). The results confirmed that the enhancer of *PITPNC1* with 5 CpG sites was significantly hypermethylated by 8–16% upon PTS treatment. The enhancer region of *LINC00910*, encompassing eight CpG sites, showed 2–28% hypermethylation across eight CpG loci (Figure 3B).

### 3.4. DNMT3B Enrichment and H3K36me3 Reduced Occupancy Coincide with a Decrease in OCT1 Binding at Target Enhancers in Response to PTS

Our previous genome-wide DNA methylation study showed that 80% of regions whose DNA methylation state increases in response to stilbenoids in breast cancer cells contain a putative OCT1 binding site [20]. Most importantly, we confirmed that increased binding of DNMT3B was associated with decreased occupancy of OCT1 at the hypermethylated *MAML2* oncogene in response to PTS [20]. We therefore hypothesize that candidate genes with epigenetically targeted enhancers upon PTS are transcriptionally activated by OCT1 and OCT1 binding decreases coinciding with gene suppression in cells treated with PTS.

**Figure 4:**
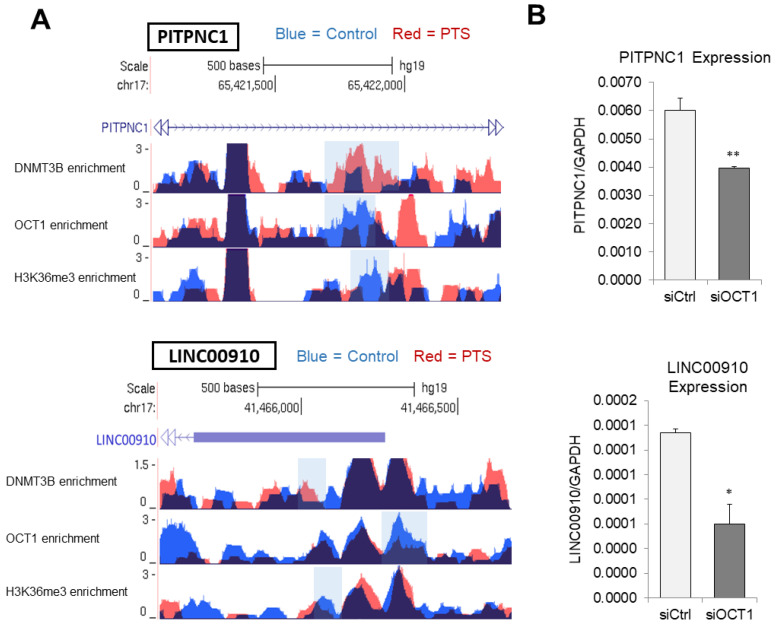
Increased DNMT3B binding and reduced H3K36me3 occupancy is associated with *PITPNC1* and *LINC00910* downregulation and hypermethylation of the enhancer regions in response to PTS in breast cancer cells. (**A**) Genome browser tracks depicting DNMT3B, OCT1, and H3K36me3 enrichment in occupancy within *PITPNC1* and *LINC00910* enhancer regions in vehicle-treated (blue, control) and PTS-treated (red) MCF10CA1a breast cancer cells. The data are based on ChIP-seq analyses. The blue-shaded region represents the DNMT3B-increased, OCT1-reduced, and h3K36me3-reduced overlapping peaks. (**B**) Expression of *PITPNC1* and *LINC00910*, as determined by QPCR in MCF10CA1a breast cancer cells with OCT1 knockdown, which was generated using siRNA. Cells transfected with scrambled siRNA (siCtrl) served as a control. All results represent mean ± SD of three independent experiments; ** *p* < 0.01, * *p* < 0.05.

To understand these events, we performed ChIP sequencing following ChIP with OCT1-specific antibody. Among 106 peaks that were characterized by increased DNMT3B binding and reduced H3K36me3 mark and located in regions relevant to transcription (Figure 2A), 46% overlapped within 200 bp with an OCT1-reduced peak. Six peaks out of seventeen enhancer regions where epigenetic marks changed upon PTS (i.e., DNMT3B and H3K36me3) contained an overlapping OCT1-reduced peak (Supplementary Table S2). Genome browser tracks of the ChIP-seq data in Figure 4A show the overlap in increased DNMT3B enrichment, reduced OCT1 binding, and reduced H3K36me3 occupancy at enhancer regions of *PITPNC1* and *LINC00910*, in MCF10CA1a cells treated with PTS (red peaks) as compared with control MCF10CA1a breast cancer cells (vehicle-treated, blue peaks).

To determine the role of OCT1 in the regulation of *PITPNC1* and *LINC00910* transcription, we tested the expression of the genes in MCF10CA1a cells with siRNA-mediated OCT1 depletion, which were generated in our previous study [20]. OCT1 depletion led to a profound downregulation of the two tested genes (Figure 4B). It indicates that OCT1 plays a role in driving the expression of *PITPNC1* and *LINC00910*.

## 4. Discussion

A crucial role of enhancers in gene transcriptional activity has long been an area of interest, with a more recent focus on the contribution of epigenetic components, including DNA methylation, in regulating the activity of enhancer regions [10,14,15]. Dysregulation of DNA methylation patterns and aberrant expression of DNMTs have been observed across multiple cancer types [59,60]. For instance, tumor suppressor genes are often methylated and silenced during carcinogenesis, whereas oncogenes lose methylation within their regulatory regions, including enhancers, and become actively transcribed [5,20]. Our group has shown that stilbenoids mediate transcriptional repression of *MAML2* through altering epigenetic patterns at the enhancer region of this oncogene [20]. DNMT3B was suggested to be a key player in *MAML2* hypermethylation and silencing in response to stilbenoids.

In the present study, we therefore investigated epigenetic marks and the interaction of DNMT3B with DNA at enhancer regions in response to PTS in breast cancer cells. Following ChIP-seq, we found 173 DNMT3B-increased peaks within enhancer regions corresponding to 77 unique genes (Figure 1D and Figure 2A). Functional analyses show enrichment of these genes with oncogenic processes and pathways, for example, *PITPNC1* is involved in metastasis [46], *NOTCH2NL* is from the NOTCH signaling pathway [40], and *SREBF1* is a transcription factor linked to oncogenic PI3K/AKT/mTOR pathway [51]. Of importance, although DNMT3B’s role in the regulation of gene transcription appears to be context-dependent, the enzyme has previously been shown to target genes with oncogenic functions for methylation and silencing in different cancers, including breast cancer [61,62,63,64]. Interestingly, in our ChIP-seq data, reduced binding of DNMT3B in response to PTS seems to occur at different genes than the increased binding. Among genes associated with DNMT3B-reduced peaks within gene regulatory regions, we found strong candidates associated with inhibition of cancer proliferation, migration, and metastasis, and activation of apoptosis. For instance, we identified *CHRDL1*, the downregulation of which was associated with poor survival and mechanistically with cancer progression and metastasis, indicating the tumor-suppressing role of *CHRDL1* [65]. Other interesting candidates were *NOX5*, whose activation was demonstrated to inhibit cancer stem cell formation through ROS generation [66], and *SALL3*, which was reported to directly inhibit DNMT3A activity and consequently cause DNA hypomethylation and activation of tumor suppressor genes [67]. Thus, our findings imply that DNMT3B may be implicated in the methylation and silencing of oncogenes in normal cells.

Interestingly, previous studies report delocalization of DNMT3B in cancer and link this phenomenon, at least partially, to oxidative stress and DNA repair mechanisms [68,69]. It is suggested that oxidative stress leads to DNA breaks and activation of the DNA repair pathway, which consequently recruits DNMT3B to sites of DNA damage [68,69]. When the repair system is overloaded, it may cause a permanent shift in DNMT3B localization, resulting in a transition from the silencing of oncogenes to the methylation and silencing of tumor suppressor genes. As PTS is known to regulate the antioxidant defense network [70,71,72], we could hypothesize that PTS, by decreasing oxidative stress, may alter DNMT3B localization and shift it back to regions where DNMT3B typically binds in normal cells [22]. This interesting concept will be further explored in our future studies.

We found that 17 of the enhancer regions with DNMT3B-increased binding in response to PTS overlap within 200 bp with reduced occupancy of H3K36me3 (Figure 2). H3K36me3 is a very important histone mark linked to transcriptional activation and elongation and distinguishing active from intermediate or poised enhancers [13]. H3K36me3 was found to be abundant along with phosphorylated RNA polymerase II at actively transcribed genes, and, as such, it is an excellent mark of transcribed genes [13]. Nearly 31% of genes with DNMT3B-increased peaks in regions relevant to transcription lost H3K36me3 in response to PTS (Figure 1D). This clearly indicates a decrease in the transcriptional activity of those genes upon PTS.

Genes assigned to the 17 enhancers included oncogenes and pro-metastatic genes (*PITPNC1* [46], *TNNT2* [47], *ZP4* [48], *CUX1* [55], *RYR2* [57,58]), and putative oncogenes (*DANT2* and *LINC00910*). Indeed, enrichment of ectopic H3K36me3 at oncogenes has previously been reported in different cancers, including breast cancer, and associated with gene upregulation [73,74,75,76,77]. Redistribution of H3K36me3 across intergenic regions and low-expressing genes was associated with aberrantly upregulated expression [78]. As H3K36me3 is known to antagonize Polycomb repressive complex 2 (PRC2)-mediated H3K27 methylation [79,80], perhaps loss of H3K36me3 at enhancers of oncogenes in response to PTS results in increased PRC2-mediated gene repression. Another interesting aspect is that genes expressed in a cell type-dependent manner are known to be targeted by de novo DNMT activity and methylated as a consequence [81]. DNA methylation eventually replaces dynamic histone marks to provide stable silencing of these genes upon differentiation [81]. Indeed, along with increased occupancy of DNMT3B, we observed DNA methylation and decreased expression of *PITPNC1* and *LINC00910* (Figure 3).

In addition, almost half of the overlapping DNMT3B-increased and H3K36me3-reduced peaks located in regions relevant to transcription (i.e., promoters, enhancers, insulators, Polycomb-repressed regions, or heterochromatin) also lost OCT1 binding in response to PTS (Figure 4A, Supplementary Table S2 with enhancers). This finding raised the possibility that OCT1 plays a role in the regulation of transcription of corresponding genes as all the changes refer to reduced transcription. Indeed, OCT1 depletion in MCF10CA1a breast cancer cells led to a profound downregulation of *PITPNC1* and *LINC00910* (Figure 4B) and exerted anti-cancer effects [20]. Taking into account that the overlapping DNMT3B-increased and H3K36me3-reduced peaks within regions relevant to transcription are assigned to genes enriched with cancer-driving functions and pathways (Figure 1C), our results are in agreement with existing literature. The pro-tumorigenic function of OCT1 has been demonstrated in different cancer types, and OCT1 has been shown to regulate genes associated with cell metabolic function, proliferation, oxidative stress, and immune modulation, all of which are interconnected with a process of tumorigenesis [82,83]. DNMT3B- and OCT1-mediated mechanisms, through which stilbenoids modify DNA methylation and impact the expression of genes involved in cancer, are an emerging research area.

The overlap between OCT1-reduced peaks and DNMT3B-increased and H3K36me3-reduced peaks also suggests that OCT1 may matter for specificity in DNMT3B binding to those regions. Indeed, several pieces of evidence indicate that DNMT3B recruitment may be directed by the recognition of transcription factors [39,84]. It was reported that certain transcription factors, for example, E2F6, NR6A1, and PU.1, act as positive regulators of DNMT3B recruitment, leading to silencing of their target genes [84], whereas CTCF and SP1 were shown to block de novo DNA methylation at the target regions [84].

In terms of DNMT3B recruitment to the target regions, another interesting hypothesis involves histone modifications, such as H3K36me3, as was suggested by other groups [85,86]. DNMT3B selectively bound the bodies of transcribed genes and led to their preferential methylation, which was mediated through recognition of H3K36me3 in mouse embryonic stem cells [85]. Recruitment of DNMT3B to cell-type-specific actively transcribed enhancers, followed by their hypermethylation, was also demonstrated to be mediated by recognition of H3K36me3 in human epidermal stem cells [86]. Depletion of SETD2, an enzyme responsible for H3K36me3, particularly affected DNA methylation at H3K36me3 sites, implying that H3K36me3 is key to guide loci-specific recruitment of DNMT3B [85]. On the other hand, as we mentioned before, loss of H3K36me3 may result in increased PRC2-mediated gene repression [79,80]. Therefore, the increase in DNMT3B binding and DNA methylation within regions of decreased H3K36me3 upon PTS may just be a consequence of decreased expression, irrespective of H3K36me3 recruitment activity of DNMT3B. Those interesting interactions between epigenetic enzymes and proteins and the consequences of such interactions for the regulation of gene expression should be further explored in vivo. Of importance, current technological advances in cell sorting allow for the isolation of single cells from tumors [87,88,89]. It makes the in vivo approach highly feasible. It would also be critical to elucidate whether DNMT3B plays similar roles in other cancers, especially other aggressive cancers characterized by activation of oncogenes (e.g., pancreatic cancer [90]), and whether PTS effects on DNMT3B binding and histone mark occupancy occur in different cancer types.

## 5. Conclusions

In summary, our study delivers new knowledge about the effects of dietary polyphenols on epigenetic marks and the interaction between DNA and epigenetic enzymes at enhancer regions. We show that PTS-mediated silencing of known or putative oncogenes involves the interaction between DNMT3B, H3K36me3, and OCT1, which opens the door to further investigations. Such epigenetic silencing of oncogenes may be an important contributor to the anti-cancer action of stilbenoid polyphenols. Insights into the mechanistic underpinnings of bioactive compounds eliciting anti-cancer effects are crucial in the development of effective cancer prevention strategies and novel approaches in support of existing therapies.

## Data Availability

GEO accession number: GSE175639.

## Supplementary Materials

The following are available online at https://www.mdpi.com/article/10.3390/antiox10081232/s1, Table S1: Pyrosequencing and QPCR primer sequences for *PITPNC1* and *LINC00910*, Table S2: Overlapping peaks between DNMT3B increase, H3K36 decrease, and OCT1 reduction within enhancer regions.
