# Supplementary material for: Pterostilbene Changes Epigenetic Marks at Enhancer Regions of Oncogenes in Breast Cancer Cells
Source: Antioxidants (Basel). 2021 Jul 30;10(8):1232. doi: 10.3390/antiox10081232 (PMC8388921; doi:10.3390/antiox10081232)
Supplement: Supplementary file 1 [file antioxidants-10-01232-s001.zip › Supplementary Table S1.pdf]

Supplementary Table S1: Pyrosequencing and QPCR primer sequences for *PITPNC1* and *LINC00910*.

Gene	Primer sequences	Annealing temperature [°C]	Amplicon length [bp]
**Pyrosequencing**			
PITPNC1 (CpG #1-5)	FW 5'- AGGAATAGTTTGAATTTGGGAGG -3'<br>RVBio 5'- AACCTCTACAACCTACTTATTAACTACAT-3'<br>Seq 5'- AGGAGAAGGTTGTAGTGA -3'	58.5	181
LINC00910 (CpG #1-8)	FW 5'- TTGAGGGTTGGGATTTTTATTAGTAT -3'<br>RVBio 5'- ACCTCCTAACTACCTCCTCTCTAATTAC -3'<br>Seq 5'- AGTGTTTGGTAAGTTGA -3'	58.5	165
LINC00910 (CpG #9-15)	FW 5'- TTAGAGAGGAAGTAGTTAGGAGGTTATTGG -3'<br>RVBio 5'- CTCAAAAAAAAAATTTATCCCAACCTTAC -3'<br>Seq 5'- GTTAGGAGGTTATTGGTT -3'	55	165
**QPCR**			
PITPNC1	FW 5'- GGACAACAAAGGAAGCAATGAC-3'<br>RV 5'- TTGTAGTAGCGCTCTGGAATTT-3'	59	111
LINC00910	FW 5'- ACGTTCACAGGTACACAAAGG -3'<br>RV 5'- TCCCAGTATCCGACTAGCTTC -3'	59	92
GAPDH	FW 5'-TGCACCACCAACTGCTTA-3'<br>RV 5'-AGAGGCAGGGATGATGTTC-3'	59	177
